# *Toxoplasma gondii *peroxiredoxin promotes altered macrophage function, caspase-1-dependent IL-1β secretion enhances parasite replication

**DOI:** 10.1186/1297-9716-42-80

**Published:** 2011-06-27

**Authors:** Edward S Marshall, Hany M Elshekiha, Mohamed-Ali Hakimi, Robin J Flynn

**Affiliations:** 1 School of Veterinary Medicine & Science, Faculty of Medicine and Health Sciences, University of Nottingham, Sutton Bonington Campus, Leicestershire, LE12 5RD, UK; 2 Laboratoire Adaptation et Pathogénie des Micro-organismes, Université Joseph Fourier Grenoble 1, BP 170, F-38042 Grenoble cedex 9, France

## Abstract

Alternatively activated macrophages (AAM) are a key feature Th2 immunity and have been associated with a variety of roles during helminth infection. The role this cell subset plays in protzoan infection remain relatively unexplored, herein we describe the effects of a redox enzyme (rTgPrx) derived from *Toxoplasma gondii *on murine macrophage phenotype in vitro. RTgPrx has been previously associated with the maintainence of parasite oxidative balance. Here our experiments show that rTgPrx promotes AAM as indicated by high arginase-1 (arg-1), YM1 and FIZZ expression via both signal transducer and activator of transcription (STAT)6-dependent and -independent mechanisms. Additionally rTgPrx treatment reduced caspase-1 activity and IL-1β secretion, while simultaneously increasing IL-10 release. Furthermore the in vitro replication of *T. gondii *(RH strain) was enhanced when macrophages were treated with rTgPrx. This is in contrast with the previously described effects of a *Plasmodium berghei *ANKA 2-cys-peroxiredoxin that promotes pro-inflammatory cytokine production. These results highlight the role of *T. gondii *derived redox enzymes as important immune modulators and potentially indicate a role for AAM in modulating immunopathology and promoting parasite replication during *T. gondii *infection.

## Introduction

Infection with the protozoan parasite *T. gondii *can occur via the oral route, the foetal-maternal interface, or by consumption of undercooked meat containing parasitic cysts [1]. Ovine toxoplasmosis is a major loss to the agricultural industry through foetal loss The outcome of infection for the unborn lamb during pregnancy is dependent on whether infection takes place during early/mid-gestation or late gestation leading to death or live birth, respectively. At present a commercially available live vaccine is on the market and is effective at preventing congenital infection in ewes if administered before pregnancy. Infection via the oral route results in a highly polarised Th1 cell response, where this response is uncontrolled it leads to CD4+ dependent mortality [2]. In response to the strong Th1 response the parasite forms long-lived tissue bradyzoites which can become reactivated in immunocompromised individuals. Reactivation of the cysts within give rise to toxoplasmic encephalitis (TE) resulting in neuropathology [3]. Parasite and host survival is mediated by control of the host immune response through a combination of mechanisms; these can include generation of T-regulatory cells [4], and the production of parasite immunomodulators [5]. *T. gondii *possesses an arsenal of secreted or injected proteins that can modulate host cell function. Injection of rhoptry bodies into host cells allows efficient entry and replication of the parasite [6] and modulation of type-1 immune response genes [7]. Induction of cellular autophagy is a host defence mechanism by which parasite replication can be controlled, however it has recently been shown that more virulent strains of the parasite may also be capable of subverting this pathway [8].

Macrophages sit at the bridge between innate and adaptive immunity and they serve a number of functions, during protozoan infection controlling parasite replication is chief amongst them. Modulation of macrophage functional status to either of the two defined phenotypes classically activated macrophages (CAM) or AAM [9] by the parasite may serve to modulate host immunity and prolong parasite survival. A number of defined *T. gondii *pathways exist have been shown to very specifically modulate host immunity including macrophage functions and pathways such as STAT and TLR signalling [10].

Helminth peroxiredoxins (Prx) derived from the flukes *Schistosoma mansoni *and *Fasciola hepatica *has been shown to drive the activation of AAM [11]. These cells display the characteristic markers of AAM including upregulated arginase-1 (arg-1), Ym1, FIZZ and also expressed IL-10. Interestingly in the case of *F. hepatica *Prx these effects have been shown to be independent of enzymatic activity in mouse and ovine macrophages [11,12]. This is highly suggestive of a dual function for this class of molecules in helminth parasites. Furthermore the development of AAM has also been linked with enhanced survival of intracellular pathogens during a co-infection. *Taenia crassieps *infected mice which are subsequently infected with *Leishmania major *and *L. mexicana *display higher *Leishmania *burdens and these are localised to AAM within the tissues of these mice [13]. Recently a 2-cys Prx from *P. berghi *has been shown to trigger TLR4 on periteonial macrophages resulting in the release of pro-inflammatory cytokines such as tumour necrosis factor (TNF)-α and IL-12p40 [14] these results contrast with the described effects of helminth anti-oxidants. A *T. gondii *Prx 1 (TgPrx) has been cloned [15], and studies have shown that it interacts with a novel *T. gondii *histone lysine methyltransferase (KMTox) [16]. This complex was shown to regulate parasite genes related to antioxidant defences and the maintenance of cellular homeostasis, defining it as a functioning anti-oxidant.

Despite the role of TgPrx in maintaining cellular homeostasis, it is unknown if TgPrx has a function in the context of host macrophage activation either equal to that of *P. berghi *or helminth Prx. Here we have investigated what if any effect recombinant (r)TgPrx had on macrophage functions or effector mechanisms. In this study we provide evidence that rTgPrx can induce murine AAM markers and enhance secretion of IL-10. We also found that rTgPrx could negatively influence the secretion of IL-1β from macrophages. Ultimately resulting in rTgPrx promoting parasite survival as judged by replication of *T. gondii *in treated cells. This is highly suggestive that rTgPrx may have acquired/developed additional roles during infection related to the modulation of host macrophage function.

## Materials and methods

### *T. gondii *strain, culture, and infection of macrophages

*T. gondii *RH strain was obtained from the ATCC and maintained in VERO cells as previously described [17]. After 4 days of infection confluent monolayers of host cells were collected and parasites harvested for infection of macrophages. For parasite survival assays, host cells were seeded onto coverslips (Nunc) overnight. Prior to infection cells were treated overnight with LPS, IL-4 or rTgPrx as described within the text or below. Parasites were used at a multiplicity of infection (MOI) of 0.2, 24 hrs following infection coverslips were stained with Giemsa and the numbers of parasites were counted on a high power view in 100 cells per treatment.

### Recombinant *T. gondii *Prx

*T. gondii *Prx coding sequence, accession number 305718, as described elsewhere [15] was used to prepare recombinant protein [16]. In brief, the coding sequence was cloned, in frame, into an expression vector and tagged at the C-terminus with a His6 tag. This plasmid was expressed in *E. coli *to produce recombinant proteinthat was purified using nickel affinity chromatography. Purified protein was dialysed extensively against sterile endotoxin-free 1 × D-PBS overnight at 4C. Protein (200 ug) was then phase separated, as previously described, to remove contaminating endotoxin [18]. Briefly, protein was mixed with Trition X-114 to a final concentration of 5% and incubated on ice for 5 min and then at 37°C for 5 min. Solutions were centrifuged at 5000 × *g *for 7 s to form two phases. The upper aqueous phase contained endotoxin free protein. This processed was repeated with the aqueous phase. Following this the protein was again dialysed as above and protein concentration was determined using a Nano-drop. Endotoxin levels as judged by LAL (Lonza) endpoint assay were below the limits of detect of the assay.

Enzymatic activity was determined by use of rTgPrx in a DNA nicking assay [19]. rTgPrx was incubated with DTT (8 mM) and FeCl3 (0.8 mM) for 15 min prior to adding 300 ng of DNA prepared from the pGEM-T vector (Promega). As a negative control DTT was omitted or proteinase K treated rTgPrx was used. Reactions were incubated for 2.5 h at 37°C and then visualised on a 1% agarose gel. Enzymatic activity was denoted by DNA being protected from nicking.2.3 Macrophage culture and stimulation.

Bone marrow derived macrophages (BMDM) were generated by culturing cells collected from the femurs of C57/bl6 mice with M-CSF (Peprotech), 10 ng/mL, supplemented media for seven days with a change of media on day 3. Recombinant IL-4 (Peprotech) was used at 20 ng/mL, LPS (Sigma-Aldrich) was used at 100 ng/mL, and rTgPrx was as either as indicated or 80 μg/mL. For activation caspase-1 activation and IL-1β secretion monosodium urate, MSU, (Sigma-Aldrich) was used at a concentration of 150 μg/mL. Following 24 h stimulation supernatants were collected, centrifuged at 4°C to remove debris and stored until analysis. Cells were lysed in 0.1% Trition X-100 for analysis of arginase activ. For later determination of caspase-1 activity 50 μL of cell lysis buffer (ENZO Lifesciences) was added to each well. To inhibit STAT6 activity leflunomide (Sigma-Aldrich), at a concentration of 100 μM, was added to cells 30 min prior to treatment.

### Arginase activity, NO, and cytokine measurement

Arginase activity was measured in cell lysates as previously described and reported as mU/106 cells [20]. NO concentration was measured using the Griess reagent system (Promega) and results are given as μM. IL-10, IL-12p40, and IL-1β were measured using ELISA kits from eBioscience according to the manufacturer's instructions, are reported as pg/mL.

### Intra-cellular ROS measurement

105 cells were seeded in a sterile 96 well fluorescence plate (Nunc) overnight to allow adherence. Following this cells were washed with HBSS and then loaded with 2, 7-Dichlorodihydrofluoresdiacetate (2, 7-DCF) (Cayman Chemical) at a concentration of 100 μM in DMEM supplemented with 1% FCS for 30 min at 37°C. Cells were washed to remove excess dye and cells were further stimulated for 60 min in HBSS with indicated proteins or cytokines. Fluorescence was then measured with excitation and emission wavelengths of 500 nm and 530 nm respectively. Results are reported as arbitrary units (AU) as experimental readings were normalised to fluorescence readings from unstimulated cells without 2, 7-DCF treatment.

### Caspase-1 activity

Following stimulation cells were lysed in cell lysis buffer (Enzo Life Sciences) 50 μL/well, this was then added to 50 μL of assay buffer (Enzo Life Sciences) containing the caspase-1 specific substrate YVAD-AFC (Enzo Life Sciences) at a final concentration of 50 nM. The reaction was allowed to proceed in fluorescence plates for 2 h at 37°C protected from light. Fluorescence was then measured at excitation and emission wavelengths of 400 nm and 505 nm, respectively. Results are reported as pmol of AFC/min/105 cells after comparison to readings taken from a standard curve generated from free AFC (Enzo Life Sciences).

### RNA isolation and gene expression

Expression of Ym1 (*chi3l3*), FIZZ1 (*retln*), and arginase-1 (arg1) genes was analysed by real-time PCR Taqman assays with reference to HPRT (*hprt1*) as a house-keeping control. Taqman assays were from ABI Systems and as follows; Mm00475988 m1 for *arg1*, Mm04213363 u1 for *chi3l3*, Mm00445109 m1 for *retlna*, and m1 for HPRT. were stimulated as above and incubated for 16 hrs, thereafter monolayers were washed once with ice-cold D-PBS (Sigma-Aldrich) and Tri-reagent (Sigma-Aldrich) was added, cell lysates containing RNA were then stored -80°C. RNA was extracted by the Phenol-Chloroform method and quantified using a Nano-drop. Reverse transcription was performed using GoScript Reverse Transcription System from Promega. Taqman assays were performed on a Roche Lightcycler. Results are reported as expression levels were calculated using the Δct method relative to HPRT.

### Statistical analysis

Data presented is the mean ± SD of triplicate measurements and is representative of at least three independent experiments. Data were analysed using GraphPad Prism and the ANOVA test. A *p *value < 0.05 was considered to be statistically significant.

## Results

### Alternative activation of murine macrophages by rTgPrx

**Figure 1:**
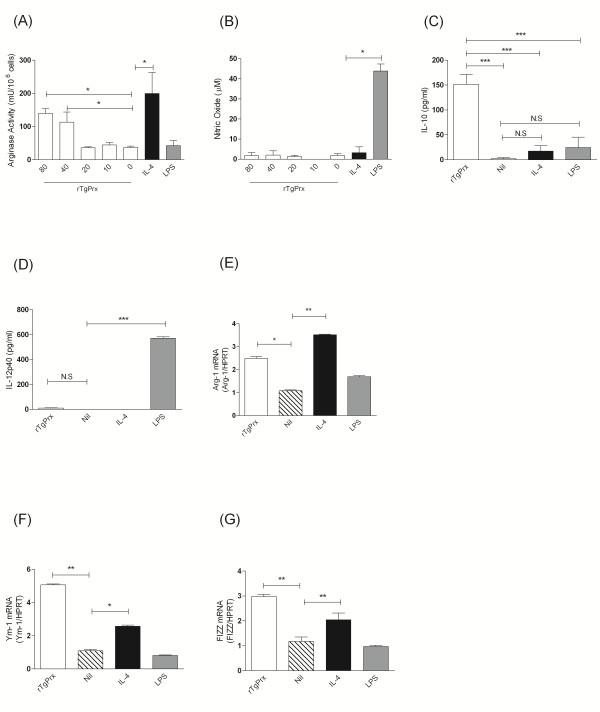
**Activation of murine macrophages by rTgPRX**. Cells were stimulated for 24 h under the indicated conditions and then analysed. (A) Cell lysates were tested for arginase activity by enzyme assay. (B) NO was measured in the supernatants from the same cultures as in (A). IL-12p40 (C) and IL-10 (D) were measured by ELISA in supernatants. PCR (E - G) was performed on cell 16 h after stimulation for FIZZ (E), Ym1 (F), and Arg-1 (G). rTgPrx at 80 μg/mL was used in panels (b), (c), and (d). *p *< 0.05 and ***p < 0.001, values represent a mean of triplicate wells ± SD; experiments were repeated three times with similar results.

BMDM were used to determine the effects of rTgPrx on macrophage activation status - cells were incubated for 24 h with increasing doses of rTgPrx. Thereafter intra-cellular arginase activity was determined; levels of arginase activity increased following treatment with either 40 μg/mL or 80 μg/mL of rTgPrx (Figure 1a). Arginase activity was not significantly different between cells treated with 40 μg/mL of rTgPrx or 20 ng/mL of IL-4. NO concentration in the supernatants of the same cells was determined and even cell treated with the highest levels of rTgPrx (80 μ/mL) produced little NO (Figure 1b). IL-12p40 considered a key pro-inflammatory cytokine produced by CAMΦ and examination of the rTgPrx stimulated cells revealed no upregulation of IL-12p40 at the protein level in comparison to LPS stimulated cells which produced 571.1 ± 20.75 pg/mL (Figure 1d). Similarly IL-10 has been linked to suppressive or AAMΦ [21]. IL-4 and LPS induced IL-10 levels above those of the controls but these were not statistically significant. However incubation of cells with rTgPrx induced roughly a 6-fold increase in IL-10 protein levels over control cultures and levels were significantly greater in comparison with other treatments (Figure 1d). We next conducted real-time to examine the expression of genes associated with AAMΦ, namely Ym1 and FIZZ1 [9]. Using IL-4 as a positive control an upregulation of FIZZ, Ym1, and arg-1 was noted in cells (Figures 1e, f and 1g). When cells were activated with rTgPrx an upregulation of these gene was also seen to occur (Figures 1e, f and 1g) suggesting a strong alternative activation profile in rTgPrx exposed macrophages. Experiments performed in cells treated with polymyxin B and rTgPrx simultaneously indicate that the above effects are not due to LPS contamination (additional file 1).

### STAT6 dependent and independent features of rTgPrx activated macrophages

**Figure 2:**
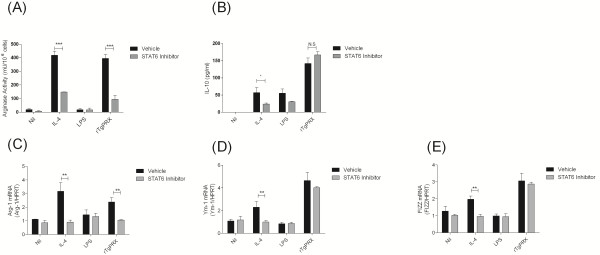
**STAT6 dependent and independent functions of rTgPrx**. Cells were treated with the STAT6 inhibitor leflunomide for 30 min prior to stimulation or with vehicle control. (A) Cell lysates were used to determine the arginase activity by enzymatic assay. (B) IL-10 levels from supernatants weremeasured by ELISA. (C, D, & E) PCR for FIZZ (C), Ym1 (D), and Arg-1 (E) was carried out on RNA isolated from stimulated cells after 16 h. ***p < 0.001 values represent a mean of triplicate wells ± SD; experiments were repeated three times with similar results.

STAT6 is a key transcription factor key to the initiation of Th2 immune responses in a number of cellular compartments, including T-cells and macrophages and acts downstream of IL-4 signalling [22]. Using a previously verified chemical inhibitor, leflunomide, of STAT6 [23] we examined whether the effects of rTgPrx were dependent or independent of STAT6. STAT6 inhibition in macrophages prior to treatment with IL-4 resulted in reduced levels of arg-1 and IL-10 protein levels (Figures 2a and 2b). Real-time PCR revealed that upregulation of FIZZ, Ym1, and arg-1 expression levels by IL-4 priming was prevented in the presence of the STAT6 inhibitor (Figures 2c-e). Treatment of macrophages with inhibitor prior to rTgPrx activation resulted in no change in IL-10 levels but a significant decrease in arginase levels (Figures 2a and 2b). Furthermore when the level of arg-1 expression was examined it too was found to be decreased in the presence of the STAT6 inhibitor. Significantly there was no reduction in Ym1 and FIZZ levels within the inhibitor treated rTgPrx activated-macrophages (Figures 2c-e). This reveals a differential control mechanism of genes involved in alternative activation induced by rTgPrx.

### Alteration of intra-cellular ROS levels and caspase-1 activation of IL-1β by rTgPrx

**Figure 3:**
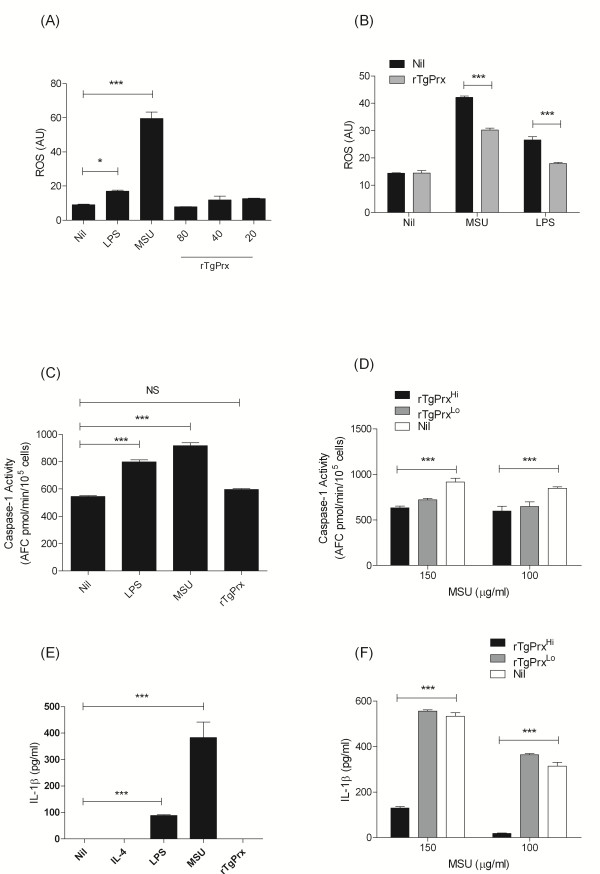
**Altered intracellular ROS and caspase-1 and secreted IL-1β levels in rTgPrx exposed macrophages**. (A & B) Cells were plated in sterile fluorescence plates overnight prior to stimulation and measurement of intracellular ROS. (C & D) Caspase-1 activity was measured in cells lysates by fluorescent enzyme assay 24 h after stimulation. (E & F) Mature IL-1β levels in supernatants were 24 determined by ELISA. *p < 0.05, **p < 0.01, ***p < 0.001, values represent a mean of triplicate wells ± SD; experiments were repeated three times with similar results.

Prx enzymes act to scavenge oxygen radicals and prevent cellular damage. Therefore, we sought to determine if rTgPrx could alter the intracellular levels of reactive oxygen species (ROS) in host cells. Using DCF labelling of cells to monitor ROS levels we found that LPS could induce ROS levels in comparison to untreated cells (Figure 3a). When rTgPrx was used in combination with LPS to stimulate cells there was no significant upregulation in ROS levels, suggesting that rTgPrx maintains an antioxidant function in the mammalian host cell environment (Figure 3b). Increases in intracellular ROS, induced by monosodium urate crystals (MSU), has been linked to inflammasome activation of IL-1β via caspase-1 cleavage of the pro-form of IL-1β. Using MSU we stimulated cells to determine if changes in ROS levels could be detected and further modulated using rTgPrx, in agreement with our previous findings using LPS we found that rTgPrx could indeed alter the ROS levels induced by MSU (Figures 3a and 3b). This effect was seen only when rTgPrx was used to stimulate cells simultaneously as MSU and when rTgPrx was at a high dose of 80 μg/mL but not at 40 μg/mL. Following stimulation with MSU we next measured intra-cellular caspase-1 enzyme activity, by fluorescent enzyme assay, and release of IL-1β. We found that MSU stimulation resulted in increased levels of both caspase-1 and IL-1β levels (Figures 3c and 3e). Using rTgPrx as an antagonist to MSU we found reductions in both the levels of caspase-1 and IL-1β release (Figures 3d and 3f). Similarly using LPS as an agonist we found that LPS can induce ROS and activate the caspase-1/IL-1β pathway and rTgPrx could act as an antagonist to this effect (additional file 2).

### Pre-treatment of host cells with rTgPrx increases parasite replication

**Figure 4:**
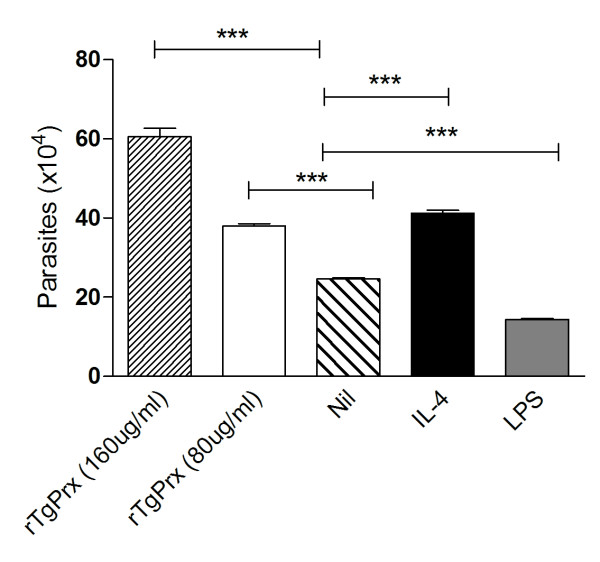
**Enhanced parasite replication in rTgPrx treated macrophages**. BMDM were pretreated with LPS, IL-4 or rTgPrx overnight prior to infection with *T. gondii*, MOI 0.2. Data represents quadruplicate counts ± SD, ***p < 0.001.

Macrophages were treated with LPS, IL-4 or rTgPrx overnight, cells were then infected with tachyzoites (M.O.I 0.2) of an in vitro adapted of *T. gondii *strain. Counts of viable parasites indicated that while LPS treated cells could control parasite replication in comparison to untreated cells, IL-4 treated cells were incapable of restraining parasite replication resulting in significant increase in parasite number compared to untreated cells (Figure 4). rTgPrx treated cells were also incapable of restraining parasite growth resulting in significantly greater levels of viable parasites compared to infected cultures of LPS treated and untreated cells, *P *< 0.001, with the greatest effect seen at a dose of 160 μg/mL of recombinant protein (Figure 4).

## Discussion

Our data presented here identifies a novel role for the *T. gondii *enzyme Prx in modulating macrophage effector functions by promoting alternative activation of macrophages and enhanced parasite replication. Exposure to rTgPrx promoted elevated arginase-1 enzyme activity and mRNA levels. In addition the markers of alternative activation Ym1 and FIZZ were also upregulated at the mRNA level. The increased expression of these genes is indicative of alternative activation [9]. Additionally IL-10 synthesis was also increased in cells treated with rTgPrx, this is a common phenotype of cells exposed to helminth molecules undergoing alternative activation [21]. We determined that the increased arginase activity caused by rTgPrx was dependent on STAT6 signalling, a key transcription factor in the initiation of Th2 responses downstream of IL-4/IL-13 signalling. However this was the only effect of rTgPrx that was STAT6-dependent, revealing differential control mechanisms of genes involved in alternative activation by rTgPrx. This is in contrast with traditional alternative activated macrophages generated by IL-4 conditioning, whereby expression of arg-1, Ym1, and FIZZ is conditional on STAT6 activation. More significantly we found that rTgPrx alters host cell ROS status suggesting that the effects of rTgPrx is reliant on intrinsic enzyme activity interfering in the host cell redox pathway to alter intracellular immunity signalling. We confirmed the need for intrinsic enzyme activity by proteinase K treatment of rTgPrx prior to experiments resulting in a loss of anti-oxidant function (additional file 3). This alteration may be responsible for our findings of reduced caspase-1 activation and IL-1β levels. Recently a report has suggested such a link between alterations in cellular redox status and activation of the NLRP3/caspase-1/IL-1β inflammasome [24]. Our final finding of increased parasite replication following pre-treatment of cells with rTgPrx indicates its role as an immunomodulator by negatively acting on anti-parasitic macrophage functions.

The function of alternatively activated macrophages within helminth parasitic infections is widely studied and they have been reported to have a number of roles dependent on model studied, including providing protection against *Heligosomodies polygyrus *infection [25], preventing immunopathology during *S. mansoni *infection [26], and controlling Th2 immunity [27,28]. However in terms of protozoan infections the role of AAM is an unresolved issue, due to the cytkine milieu that occurs during this class of infection, i.e. predominately "Th1" cytokines IL-12/IFN-γ, one might expect classically activated macrophages to dominate. There are however reports of AAM during infection with *Trypansome spp*., infection of resistant C57 mice with *T. conglesses *resulting in a changing CAM to AAM population as infection progressed [29]. This change is thought to reflect a changing need to control parasitemia initially and later to dampen inflammation and protect the host. Ultimately this switch favours host and parasite survival. To our knowledge the only work addressing the issue of AAM in *T. gondii *infection related to mice with a dominant negative mutation in the IFN-γ receptor in macrophage-lineage cells, thus making these cells incapable of activating anti-protozoan effector functions [30]. These mice despite showing the expected immune response to *T. gondii*, were unable to control parasitemia specifically within macrophages and as a consequence displayed reduced survival. Using macrophages unable to alternatively activate, non-responsive to IL-4, in the non-healing model of *L. major *infection in Balb/c mice, there was a Th2 response generated but effective control of parasite numbers within macrophages, again reflecting ineffectiveness of AAM in terms of controlling protozoan replication [31]. Taken together these results would suggest that while the presence of AAM could lead to enhanced mortality during acute infection with some protozoans, a switch to an AAM phenotype might lead to control of immunopathology and benefit both host and parasite long-term. This theory may not fit with the known course of disease following infection of ovine hosts with *T. gondii*. During pregnancy a reactivation of tissue cysts results in massive parasite replication and pathology at the foetal-maternal interface resulting in abortion [1]. The prevalent immune environment during pregnancy would favour AAM or indeed regulatory macrophages, favouring production of IL-10 and less pro-inflammatory cytokines, similar to the kind seen in our experiments. However our results certainly raise the possibility that rTgPrx may represent a previously unidentified immunomodulator.

The most pertinent questions surrounding the function of rTgPrx regards the trafficking of rTgPrx both inside the parasite and host. Analysis of the protein sequence reveals no secretion signal peptides and one must assume the enzyme is not exported from the parasite. However when initially cloned rTgPrx was found to be present in both the extra- and intra-cellular forms of the parasite. Its distribution within the tachyzoite is limited and was not found to be present in the parasitophorous vacuole or parasitophorous vacuole membrane [15]. Indeed immunolocalisation shows that rTgPrx is present in the parasite nucleus [15]. It is thought that under oxidative stress parasite signalling pathways lead to Prx translocating to the nucleus and forming a complex the KMTox, a histone lysine methyltransferase, to regulate gene expression. The question as to how rTgPrx acts on host cells will require further experimentation. However should a parasite become damaged upon entry to the host cell or as a result of oxidative damage, it is not inconceivable that cytosolic rTgPrx make "leak" into the host cell environment.

To conclude our results clearly show that an enzyme, rTgPrx, derived from *T. gondii *promotes AAM and alongside IL-10 secretion via STAT6 dependent and independent mechanisms, whilst simultaneously downregulating IL-1β production via caspase-1. Negative modulation of the inflammasome has recently been reviewed in the context of bacterial and viral infection [32], however results presented here would suggest exploration of this phenomena in the context of *T. gondii *infection warrants investigation. The functional relevance of this effect has only been explored in vitro in the context of parasite replication which is enhanced after pre-treatment of cells with rTgPrx. However the role of this protein and rTgPrx induced AAM during *T. gondii *infection warrants further in vivo investigation, the most suitable method being production of a parasite bearing a mutant Prx.

## Competing interests

The authors declare that they have no competing interests.

## Authors' contributions

ESM and RJF conceived designed and performed experiments and drafted manuscript. MAH provided recombinant proteins. HME provided helped with interpretation of results. All authors approved the final draft of the paper.

## Supplementary Material

Additional file 1 **Addition of polymyxin B to cultures containing rTgPrx does not affect alternative activation**. BMDM were stimulated in the presence of IL-4, LPS or rTgPrx in the presence of 50 mg/mL of polymyxin B. 24 h following treatment arginase activity (a), NO (b), and IL-10 (c) levels were determined. Values represent a mean of triplicate wells ± SD; experiments were repeated three times with similar results. * indicates a *P *value > 0.05 as determined by Anova. Click here for file

Additional file 2 **rTgPrx acts as an antagonist to LPS induced capase-1 and IL-1β**. BMDM were stimulated simultaneously with LPS and rTgPrx at the indicated doses. 24 h following treatment caspase-1 activity (a) and IL-1β (b) levels were determined. Values represent a mean of triplicate wells ± SD; experiments were repeated three times with similar results. * indicates a *P *value > 0.05, ** *P *value > 0.01 as determined by Anova. Click here for file

Additional file 3 **rTgPrx fails to alternatively activate BMDM following proteinase K treatment**. BMDM were stimulated in the presence PBS or rTgPrx, prior to treatment stimulants were incubated for 30 min with proteinase K. 24 h following treatment arginase activity (a), NO (b), and IL-10 (c) levels were determined. Values represent a mean of triplicate wells ± SD; experiments were repeated three times with similar results. ****indicates a *P *value > 0.001 as determined by Anova. Click here for file
