# Supplementary figures and images for: Toxoplasma gondii peroxiredoxin promotes altered macrophage function, caspase-1-dependent IL-1β secretion enhances parasite replication
Source: Vet Res. 2011 Jun 27;42(1):80. doi: 10.1186/1297-9716-42-80 (PMC3141401; doi:10.1186/1297-9716-42-80)

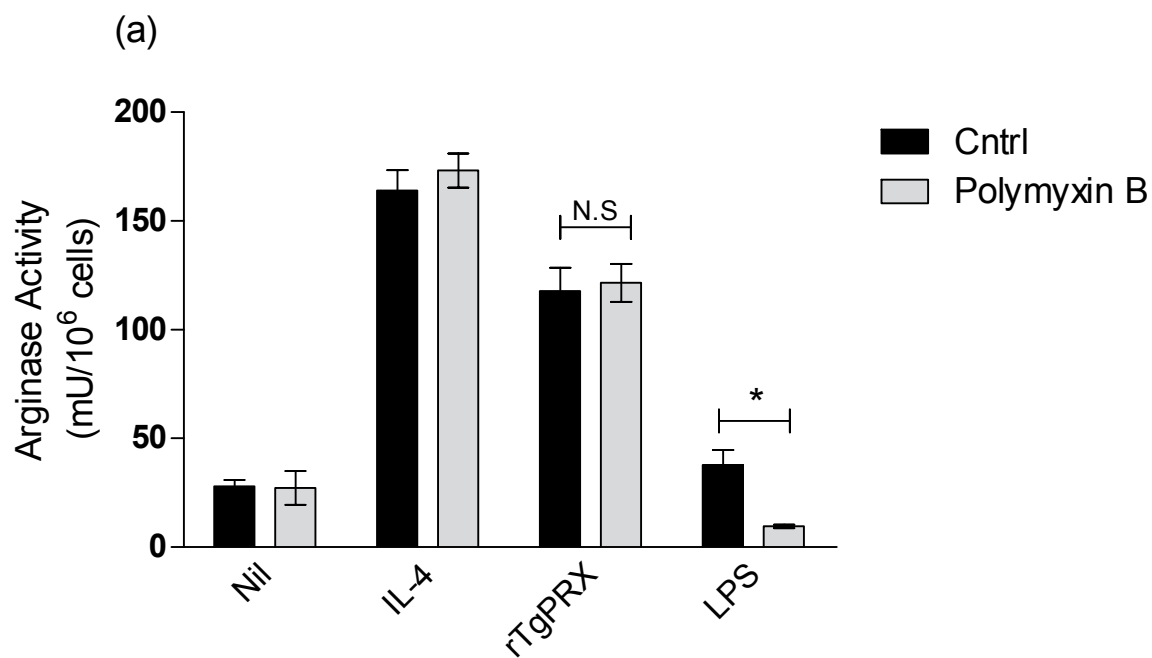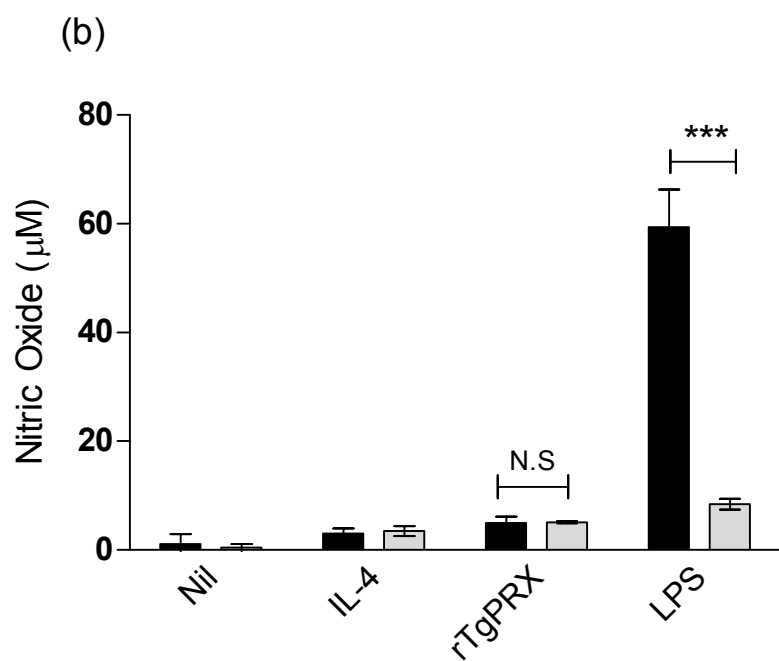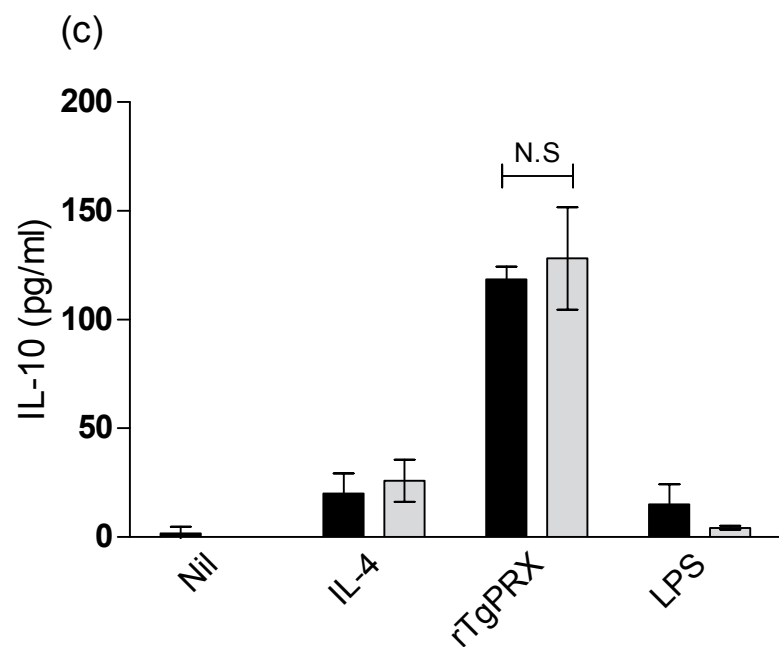

Supplement: Additional file 1 — Addition of polymyxin B to cultures containing rTgPrx does not affect alternative activation. BMDM were stimulated in the presence of IL-4, LPS or rTgPrx in the presence of 50 mg/mL of polymyxin B. 24 h following treatment arginase activity (a), NO (b), and IL-10 (c) levels were determined. Values represent a mean of triplicate wells ± SD; experiments were repeated three times with similar results. * indicates a P value > 0.05 as determined by Anova. [file 1297-9716-42-80-S1.PDF]

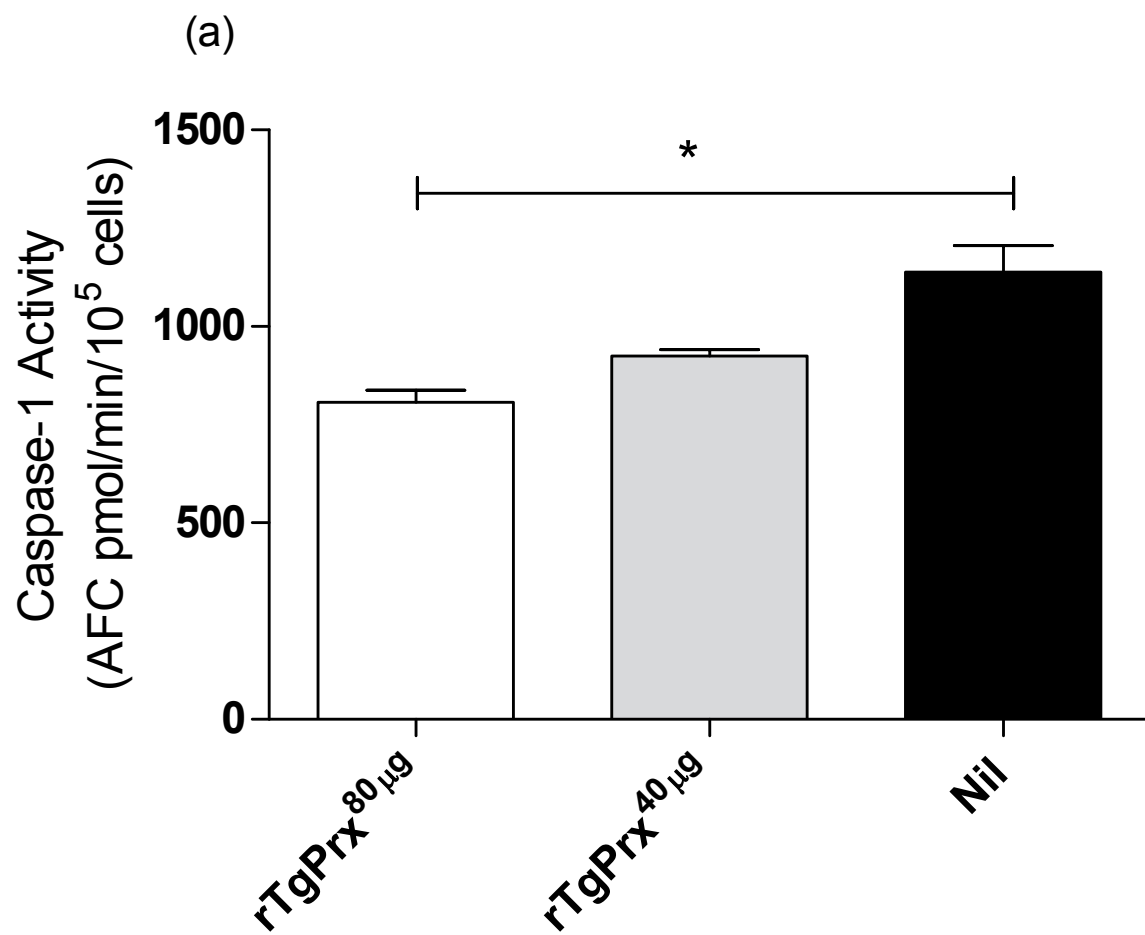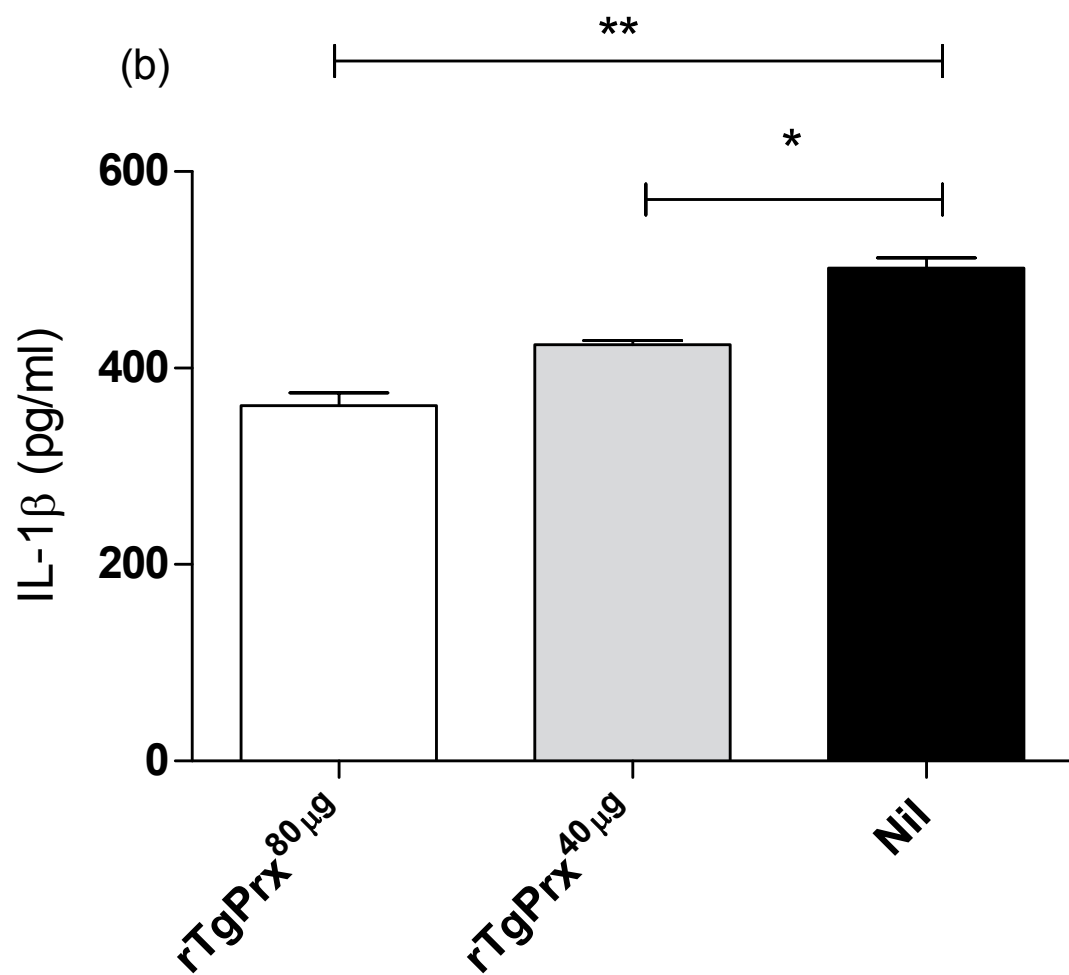

Supplement: Additional file 2 — rTgPrx acts as an antagonist to LPS induced capase-1 and IL-1β. BMDM were stimulated simultaneously with LPS and rTgPrx at the indicated doses. 24 h following treatment caspase-1 activity (a) and IL-1β (b) levels were determined. Values represent a mean of triplicate wells ± SD; experiments were repeated three times with similar results. * indicates a P value > 0.05, ** P value > 0.01 as determined by Anova. [file 1297-9716-42-80-S2.PDF]

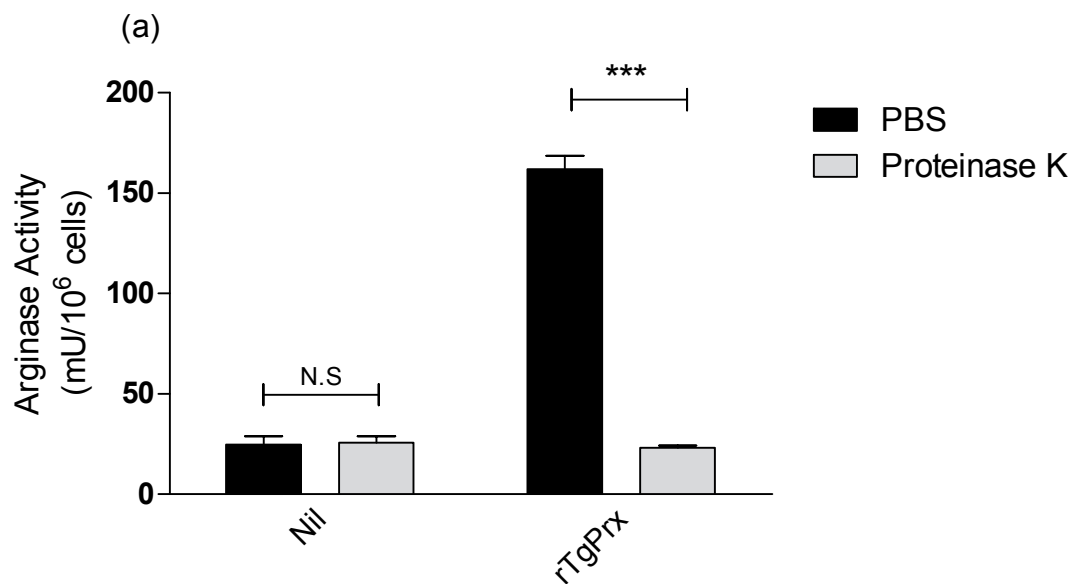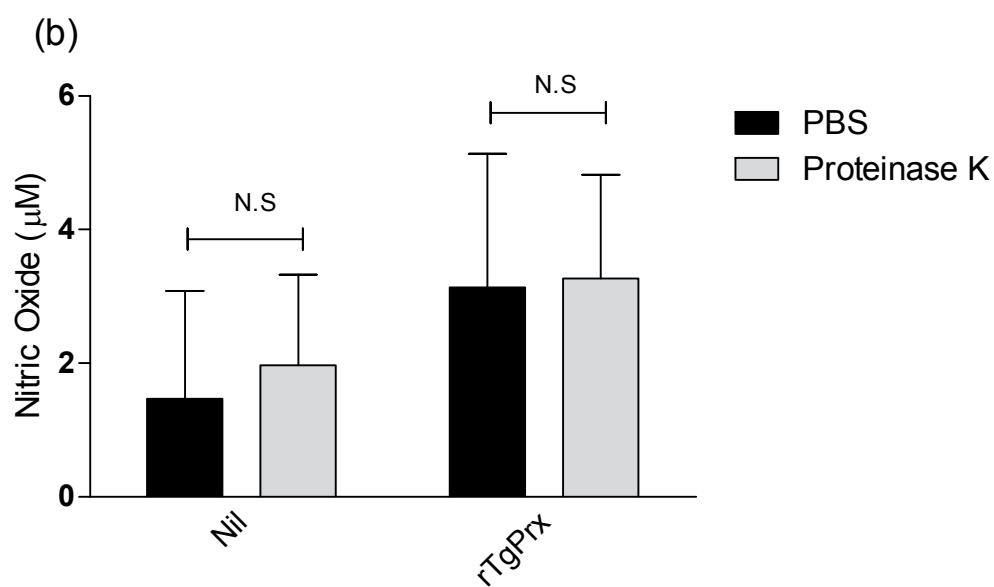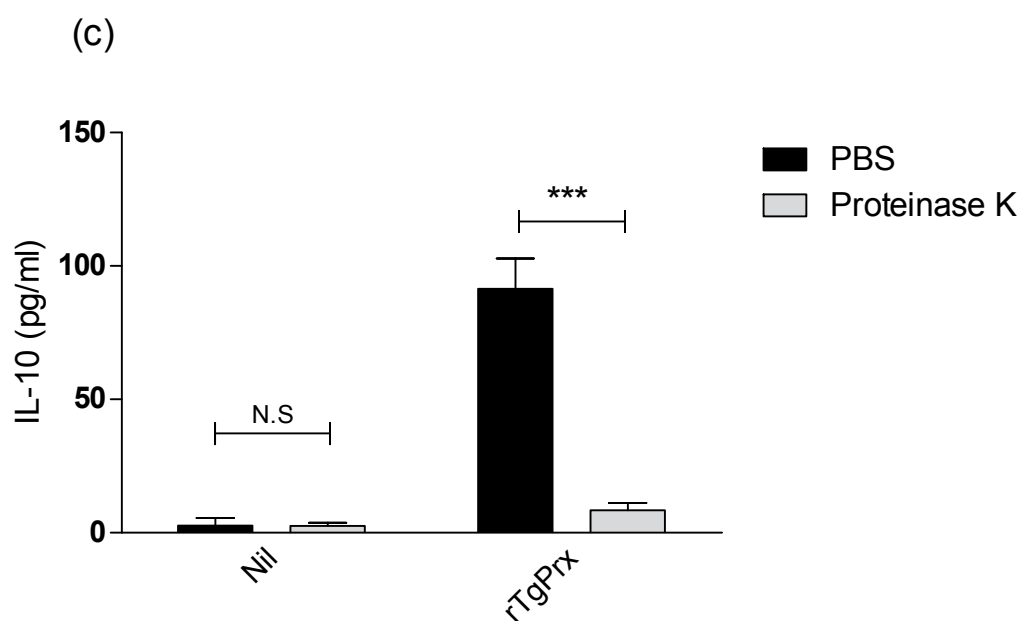

Supplement: Additional file 3 — rTgPrx fails to alternatively activate BMDM following proteinase K treatment. BMDM were stimulated in the presence PBS or rTgPrx, prior to treatment stimulants were incubated for 30 min with proteinase K. 24 h following treatment arginase activity (a), NO (b), and IL-10 (c) levels were determined. Values represent a mean of triplicate wells ± SD; experiments were repeated three times with similar results. ****indicates a P value > 0.001 as determined by Anova. [file 1297-9716-42-80-S3.PDF]
